# The Factory Nurse's Salary

**Published:** 1919-03-08

**Authors:** 


### THE EDITOR'S LETTER-BOX.

### The Factory Nurse's Salary.

*To the Editor of* THE HOSPITAL.

SIR,—I have been looking forward to the publication of the prize leaflet on "Factory Nursing" with much interest, and while the subject is most ably dealt with, I am much disappointed with the remarks *re* salary: "Factory nursing is well paid "—" Nurses seldom receive less than £100 per annum." The one remark contradicts the other, I think. If a qualified nurse is expected to give her services for £100 per annum she is, in my opinion, very poorly paid. "Welfare supervisors are paid at the rate of at least £150." Then why not nurses? Why should a nurse consider £100 a good salary under those conditions? A woman of good education who is well recommended can get an assistant welfare supervisor's post at a salary of £130 to £150, but the factory nurse with training is to be content to start at £100. Is this fair? I think not. The Welfare Supervisors' Association is looking out for its members. Who is to take care of the interests of the factory nurse? Surely she should be as well paid as an assistant welfare supervisor. In my opinion she *is* a supervisor and a very useful one.

February 25, 1919. SHELL FACTORY NURSE.

### Venereal Cases in General Institutions. A Public Danger?

*To the Editor of* THE HOSPITAL.

